# Unusual giant multilocular thymic cyst with mature teratoma including a carcinoid component in the mediastinum

**DOI:** 10.1186/s40792-022-01373-1

**Published:** 2022-01-29

**Authors:** Takehiko Manabe, Kenta Kajiyama, Takashi Iwanami, Takeshi Hanagiri, Tatsuhiko Sako

**Affiliations:** General Thoracic Surgery, Kitakyushu General Hospital, Higashijono, Kokurakita, Kitakyushu, Fukuoka 802-8517 Japan

**Keywords:** Thymic cyst, Teratoma, Carcinoid tumor, Mediastinal tumor

## Abstract

**Background:**

Teratoma is the second most common mediastinal neoplasm, but malignant transformation in mature teratomas is uncommon, and cases of carcinoid tumor with teratoma are described in only a few studies. In addition, multilocular thymic cyst associated with mature mediastinal teratoma is also a rare entity. There have been no reports of case with the coexistence of these three pathological lesions.

**Case presentation:**

The patient was a 24-year-old man who was referred to our hospital due to a 2-day history of left shoulder pain, a feeling of severe chest tightness and high fever. Pre-operative computed tomography (CT) showed a large, fluid-filled and well-demarcated multilocular cyst in the anterior to superior mediastinum measuring up to 12 cm in size. Contrast-enhanced CT also revealed that the tumor contained a solid component with slight contrast enhancement and spotty wall-thickening septation. Therefore, cystic thymoma, thymic cyst, cystic teratoma, or germ cell tumor with an inflammatory reaction were considered as differential diagnoses. The patient underwent tumor extirpation under median sternotomy. The pathological diagnosis was multilocular thymic cyst with mature teratoma including carcinoid tumor (Grade 2) in the mediastinum.

**Conclusions:**

The relationship between thymic cyst, teratoma and carcinoid tumor is unclear at present; therefore, further research is needed to clarify the relationship between these entities. In this report, we present a case of multilocular thymic cyst with mature teratoma including a carcinoid component in the mediastinum that was detected by complete surgical resection.

## Background

Carcinoid tumor arising from a mature mediastinal teratoma is very rare, with only a few documented cases. Furthermore, thymic cyst associated with mature mediastinal teratoma is also extremely rare. To the best of our knowledge, the simultaneous presence of these three pathological lesions has not been described in the literature. We herein report the first case of a patient who histopathologically presented with a multilocular thymic cyst associated with mature teratoma including a carcinoid component in the mediastinum.

## Case presentation

**Fig. 1:**
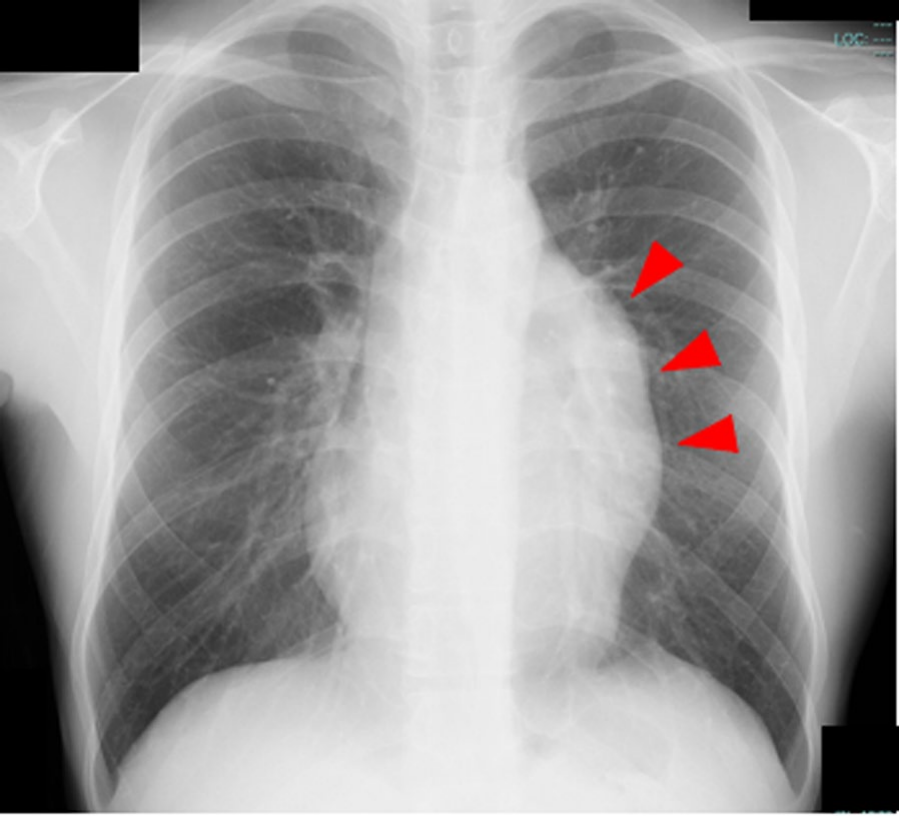
Pre-operative chest X-ray showed an abnormal mass in the left hilum region (arrowheads)

**Fig. 2:**
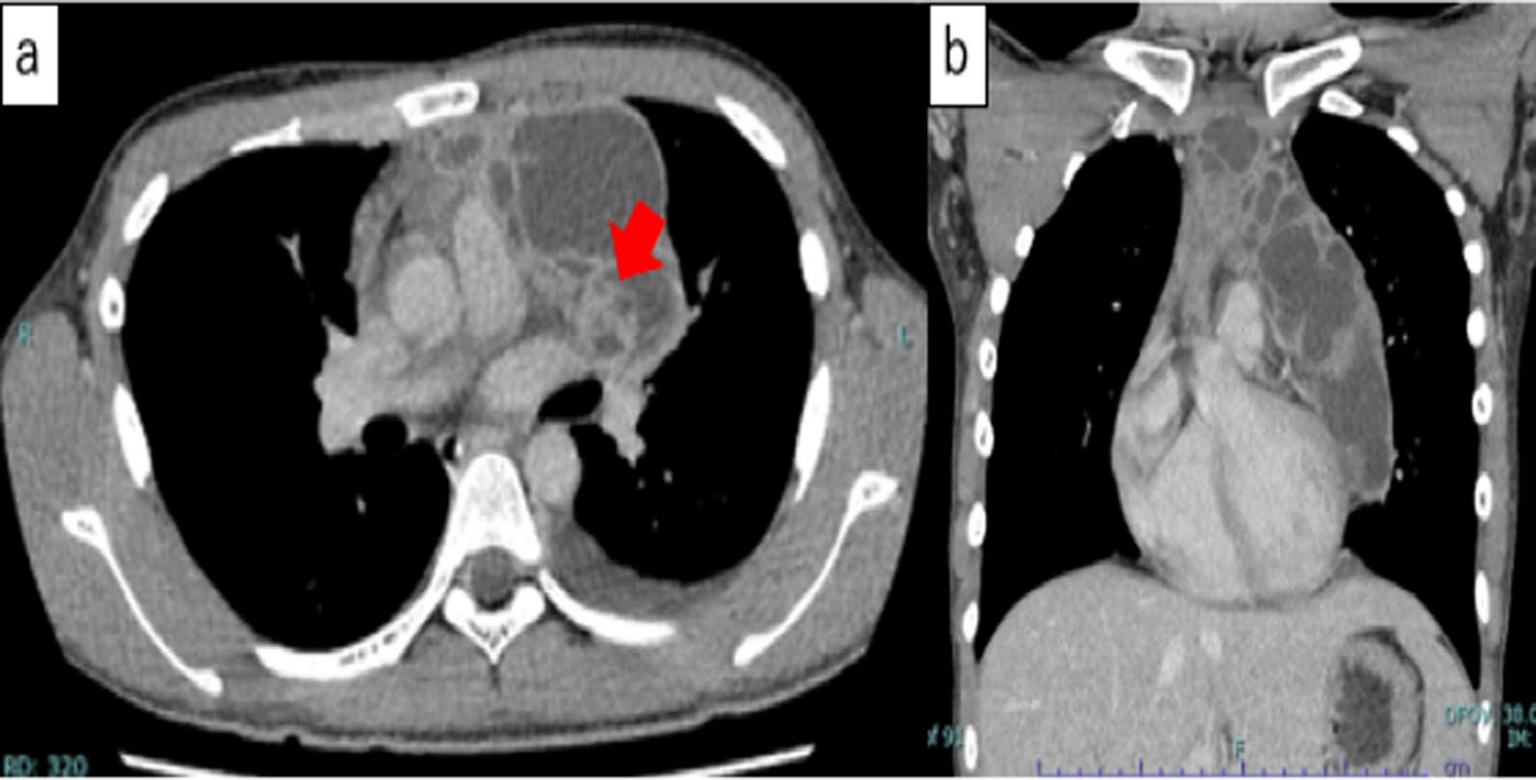
Enhanced CT revealed a multilocular heterogenous giant cyst superior to the anterior mediastinum. Septations with irregular wall thickening and a solid component (red arrow) were observed in the cyst, which had slightly higher CT attenuation than water

The patient was a 24-year-old male who presented with a 2-day history of left shoulder pain, a severe feeling of chest tightness and high fever. His family medical history was not significant, and he had not received a medical checkup for more than 5 years. Chest X-ray showed an abnormal mass in the left hilar region (Fig. 1). A blood test revealed a C-reactive protein level of 21.42 mg/dl and a white blood cell count of 14,200/µl. Tumor markers, including soluble IL-2 receptor, carcinoembryonic antigen (CEA) and alpha fetoprotein (AFP), were within the normal ranges. He had no remarkable previous medical history or history of trauma. Pre-operative computed tomography (CT) showed a large, superior to anterior mediastinal, and well-demarcated multilocular cyst. The cyst measured 12 × 10 cm in size. Septations with irregular wall thickening and a solid component were observed in the cysts (Fig. 2). Radiologically, the differential diagnoses included cystic thymoma, thymic cyst, cystic teratoma or germ cell tumor, and we considered that an inflammatory reaction in the tumor caused severe chest tightness.

**Fig. 3:**
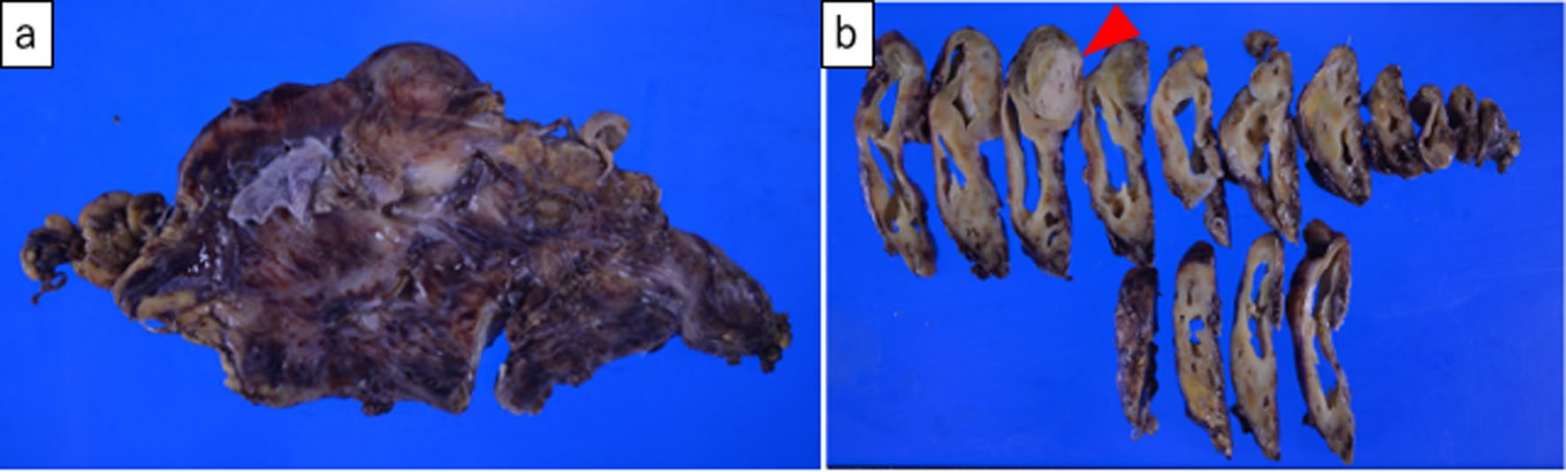
**a** Macroscopically, the nodules were 12.0, 10.0 and 6.0 cm in diameter. **b** The cut surface of the cyst revealed two distinctive parts: a greyish-white solid part (arrowheads) and a cystic part

**Fig. 4:**
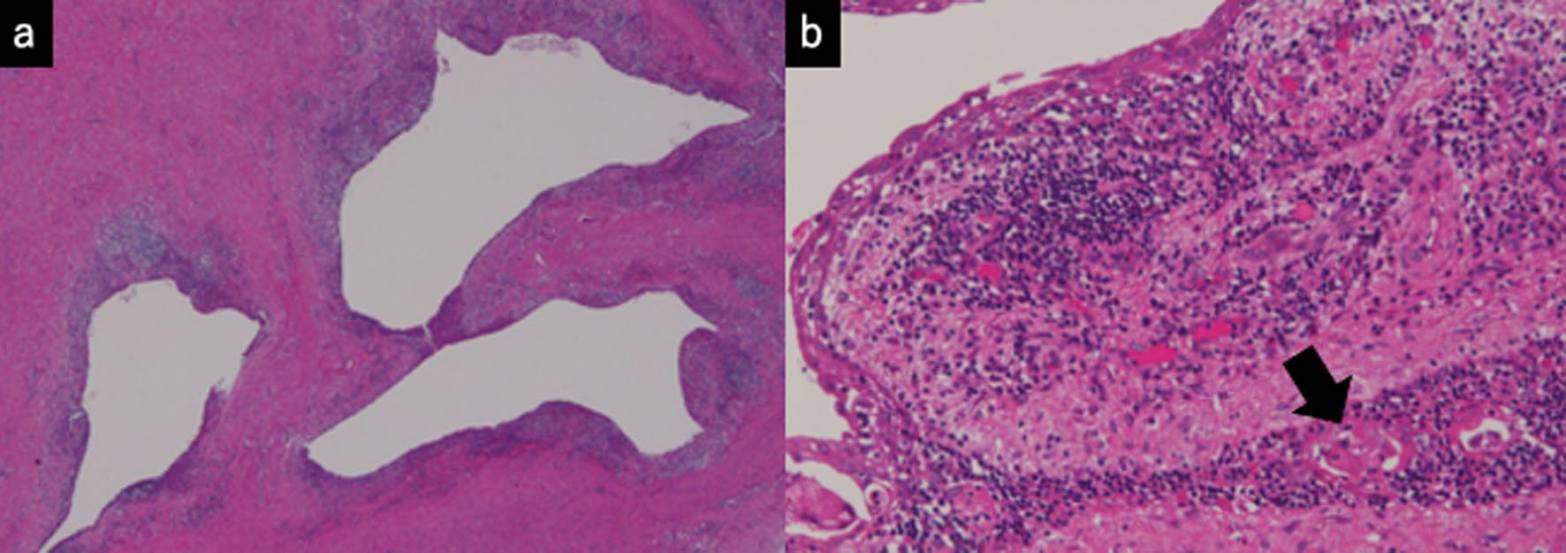
**a** Microscopically, multilocular cysts were observed in the thymic tissue. **b** The cystic spaces were lined by stratified squamous epithelium. Areas containing inflammatory infiltrates, multinucleated giant cells and Hassall’s corpuscles (arrow) were observed

**Fig. 5:**
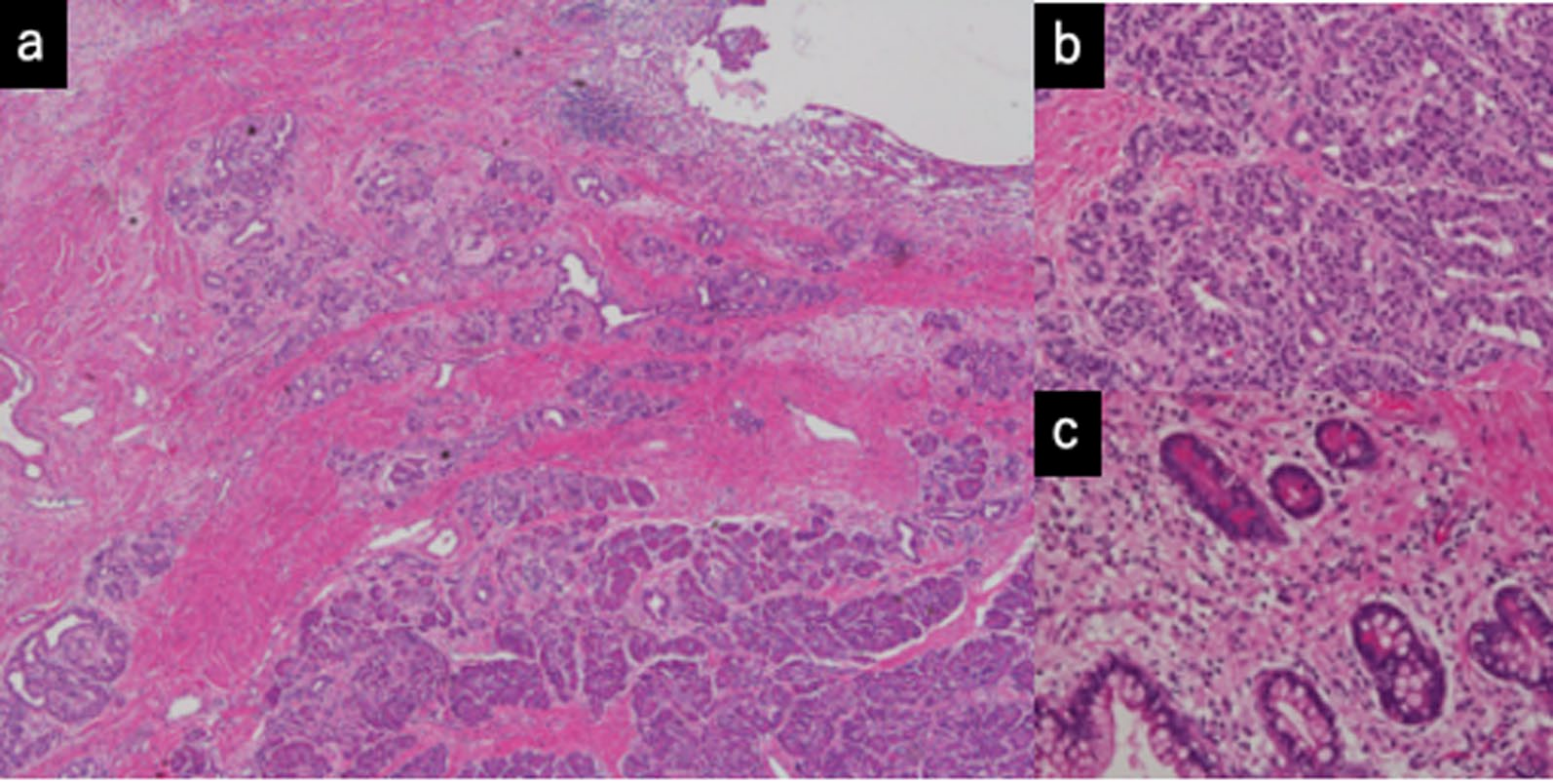
**a** Adjacent to the cystic parts, there were solid components that showed pancreatic tissues (**b**); intestinal epithelium with Paneth cells (**c**); cerebral tissues and nervous tissues

**Fig. 6:**
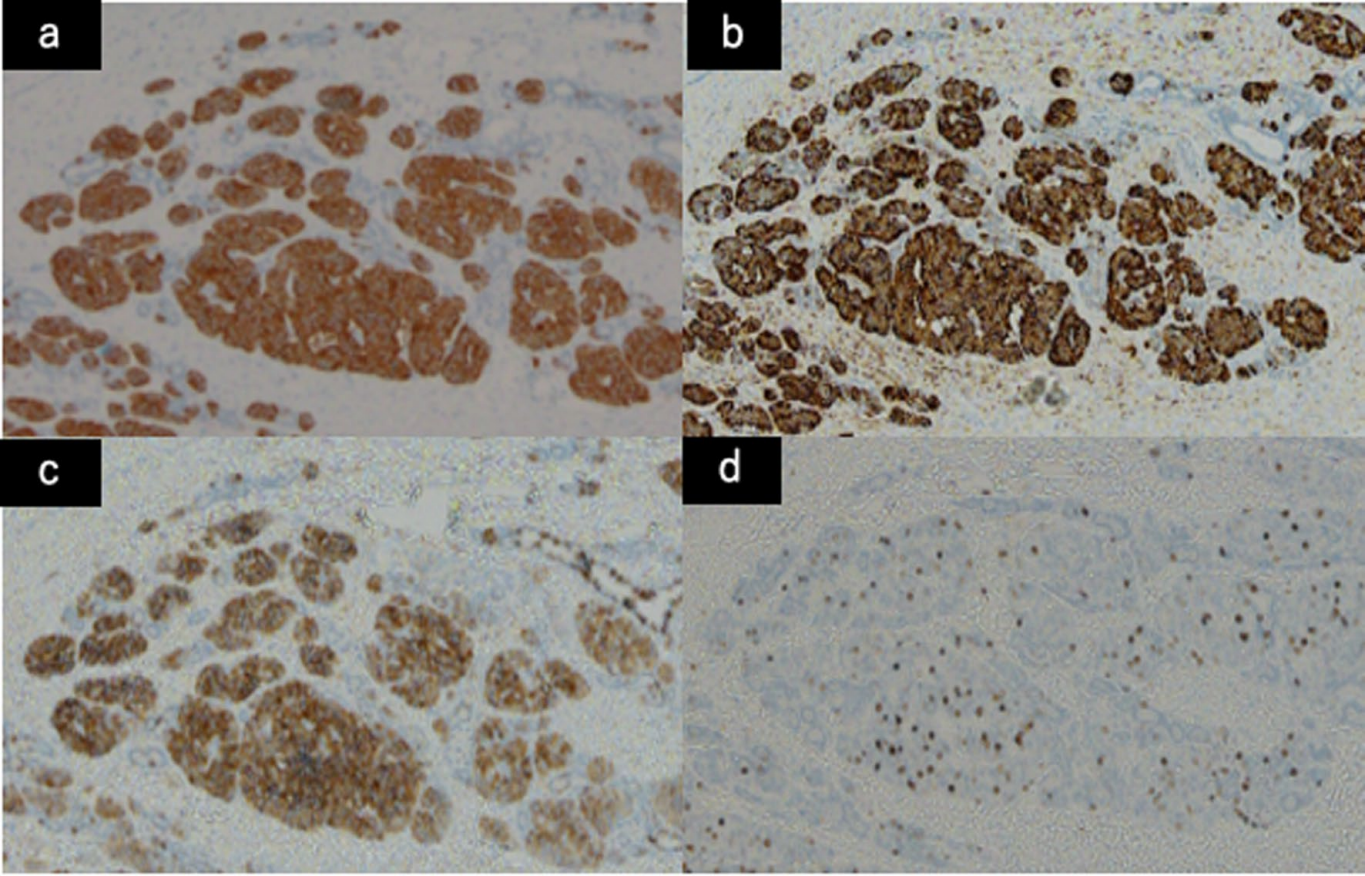
Immunohistologically, a small part of these cells was diffusely positive for synaptophysin (**a**), chromogranin A (**b**) and CD 56 (**c**), confirming neuroendocrine differentiation. The tumor had five mitoses per ten high power fields, and the Ki-67 proliferation index was 8.0% (**d**), consistent with a Grade 2

We decided to surgically remove the cystic tumor because of the possible coexistence of varied malignancies, the exacerbation of infection or the possibility of tumor rupture, including hemorrhaging or penetration to the surrounding structures. Following antibiotics treatment, tumor extirpation under median sternotomy was performed one week after the onset. During surgery, a well-encapsulated, thick-walled, and elastic hard cyst was observed in the superior to anterior mediastinum that was closed to the left brachiocephalic vein and pericardium. Since the adhesion between the tumor and pericardium was moderately strong, combined resection of the pericardium was performed and pericardium reconstruction was performed. The tumor was 12 × 10 × 6 cm in size with grayish-white discoloration (Fig. 3). The cut surface revealed a multilocular cyst in the mediastinum, which did not invade any surrounding tissues, excluding the resected pericardium (Fig. 3b). In addition, a microscopic examination of the tumor revealed that the multilocular cyst with fibrous thick-wall was lined by normal squamous epithelium cells and underlying loose connective tissue with remnants of thymic tissue (Fig. 4). Furthermore, the solid lesion of the cyst consisted of pancreatic tissues, intestinal epithelium with Paneth cells, cerebrum and nerve (Fig. 5). Immunohistologically, these cells in the solid component were diffusely positive for synaptophysin, chromogranin A and CD56, confirming neuroendocrine differentiation. The tumor had five mitoses per ten high power fields, and the Ki-67 proliferation index was 8.0%, consistent with Grade 2. No other malignancy was identified (Fig. 6).

Based on these findings, the pathological diagnosis was multilocular thymic cyst associated with carcinoid tumor (Grade 2) arising from mature mediastinal teratoma. The patient was discharged without any post-operative complications and was followed up for 1 year after surgery without any recurrence.

## Discussion

Mediastinal teratoma is a rare disease; however, it is the most common mediastinal germ cell tumor, with no significant sex difference, and can occur at any age, most commonly in individuals of 20–40 years of age. It accounts for 5–10% of all mediastinal tumors [1]. Mediastinal teratoma is derived from the spontaneous vascular development of some potential stem cells that are shed during the development of thymus primordia in the embryonic stage. It often occurs close to the thymus region [2] and usually contains tissues derived from the endoderm, mesoderm and ectoderm, and the pathological diagnosis requires at least two tissues derived from the endoderm [3]. The coexistence of a malignant component in mediastinal teratoma is extremely rare, and malignances include sarcoma, adenocarcinoma, squamous cell carcinoma and neuroendocrine neoplasms. The current terminology for these lesions, as recommended in the World Health Organization (WHO) classification, is germ cell tumor with somatic-type malignancies (GCTSM). This is defined as a germ cell tumor accompanied by a somatic-type malignant component of sarcoma, carcinoma, or both. Among GCTSM, 63% of them are associated with sarcoma [4]. In addition, some clinical studies have reported that these lesions develop in the ovary, testis, retroperitoneum, kidney, testis, mediastinum, and spinal cord. However, carcinoid tumor occurring in GCTSM, as occurred in our case, is very rare. Our search of the literature revealed only 8 cases of GCTSM with a carcinoid component occurring in the mediastinum, including the present case [4–10]. The clinical presentations included diverse symptoms, and some cases developed symptoms of infection, such as the present case.

Thymic cysts are benign entities that are classified as congenital or acquired entities in origin and are usually diagnosed in the first two decades of life. They account for only 2–3% of all mediastinal cysts [11–13]. Congenital thymic cysts are considered to be unilocular and contain clear fluid within the thin wall. On the other hand, the acquired thymic cysts are believed to be multilocular and contain turbid fluid or gelatinous material as result of hemorrhage or infection. Acquired thymic cysts are reported to be associated with thymic tumors, thymic hyperplasia, thoracostomy or chest trauma [12–14]. Inui et al. [10] described a mediastinal seminoma that coexisted with a multilocular thymic cyst. Furthermore, Dinesh et al. [15] described two cases of multilocular thymic cyst associated with mature mediastinal teratoma. However, to the best of our knowledge, the simultaneous presence of these three pathological lesions has not been described in the literature.

In the present case, the histological sections showed two distinct lesions. The first was a benign multilocular thymic cyst composed of underlying squamous cells and lymphoid tissues. Secondly, close to this cystic lesion, there was a grayish-white solid component which had various organ cells, confirming mature teratoma. In a small part of it, carcinoid differentiation (size: 6 × 3 mm) was observed. The relationship between the thymus and the tumor was unclear, although there was residual thymic tissue that existed adjacent to the mature teratoma with a carcinoid component. Complete surgical resection and a detailed histological evaluation were useful for making an accurate diagnosis and treatment, and led to a good prognosis.

Regarding treatment of mediastinal tumors, emergency surgery should be considered in cases such as ours, since the possible resulting mediastinitis and pleurisy have a high risk of causing mortality. According to the literature review by Nakajima et al., surgical resection for ruptured teratoma in mediastinum was performed 1 week after the onset of rupture in 48% of patients [16]. The sudden onset of severe symptoms, such as severe dyspnea, may suggest severe mediastinal inflammation and lead to surgeons deciding to perform surgical intervention in the early phase. However, surgical management of ruptured tumors is often more complicated than that of unruptured tumors because the internal components of the teratoma leak into the thoracic cavity, causing inflammation and adhesions. When benign teratomas cannot be excised completely without endangering vital surrounding structures, subtotal resection can relieve compressive symptoms. In our case, we performed surgery after conducting antibiotics treatment for one week. The size of the tumor, adhesion in the thoracic cavity and close distance between the tumor and left brachiocephalic vein and pericardium led to the performance of complete surgical resection combined with the pericardium through median sternotomy.

## Conclusions

We reported an extremely rare case of multilocular thymic cyst associated with mature teratoma including a carcinoid component in the anterior mediastinum, which was successfully extirpated.

## Data Availability

Not applicable.

## Abbreviations

CT: Computed tomography
LDL: Low-density lipoprotein
IL-2R: Interleukin-2 receptor
CEA: Including carcinoembryonic antigen
AFP: Alpha fetoprotein
(AFP) HCG: Human chorionic gonadotropin
MRI: Magnetic resonance imaging
